# Burs and Excavators

**Published:** 1874-11

**Authors:** 


### BURS AND EXCAVATORS.

We have for some time past been using burs for the engine and excavators, made by Spencer, Crocker and Co., that show much progress toward perfection. In variety and perfection of form they are about all that could be desired, and are well tempered. They are aiming at and rapidly approaching that point of excellence in the manufacture of the various instruments for filling teeth, for which we have long contended. We hail with great gratification every step toward excellence, that is made in the instruments and appliances that are produced for the dentist's use.

